# Phosphoinositide switches in cell physiology - From molecular mechanisms to disease

**DOI:** 10.1016/j.jbc.2024.105757

**Published:** 2024-02-15

**Authors:** Fabio Lolicato, Walter Nickel, Volker Haucke, Michael Ebner

**Affiliations:** 1 Heidelberg University Biochemistry Center, Heidelberg, Germany; 2 Department of Physics, University of Helsinki, Helsinki, Finland; 3 Leibniz-Forschungsinstitut für Molekulare Pharmakologie (FMP), Berlin, Germany; 4 Department of Biology, Chemistry, Pharmacy, Freie Universität Berlin, Berlin, Germany; 5 Charité – Universitätsmedizin Berlin, Berlin, Germany

**Keywords:** phosphoinositide, phosphatidylinositol signaling, membrane trafficking, protein-lipid interaction, lipid transport, endocytosis, exocytosis, membrane dynamics, Akt PKB, FGF2

## Abstract

Phosphoinositides are amphipathic lipid molecules derived from phosphatidylinositol that represent low abundance components of biological membranes. Rather than serving as mere structural elements of lipid bilayers, they represent molecular switches for a broad range of biological processes, including cell signaling, membrane dynamics and remodeling, and many other functions. Here, we focus on the molecular mechanisms that turn phosphoinositides into molecular switches and how the dysregulation of these processes can lead to disease.

Phosphoinositides (PIPs) are phosphorylated derivatives of phosphatidylinositol (PI), a ubiquitous phospholipid in the cytoplasmic leaflet of eukaryotic membranes that serves a plethora of functions ranging from cell signaling to membrane dynamics and host–pathogen interactions (1, 2). PI is synthesized in the endoplasmic reticulum (ER) from CDP-diacylglycerol (DAG) and *myo*-inositol by a PI synthase from where it is distributed to other organelles *via* PI transfer proteins and by membrane traffic. The *myo*-inositol moiety within PIPs assumes a “chair” conformation, with five of its six hydroxyl groups being equatorial and the one at position 2 being axial. PI serves as a membrane-bound substrate for specific kinases and phosphatases, resulting in seven known PIPs that differ with respect to their intracellular distribution and act as spatiotemporal cues to control organelle identity and to direct membrane dynamics.

### PIP synthesis and localization

PI phosphates are characterized by a relatively uniform fatty acyl composition, setting them apart from other classes of phospholipids. However, it is noteworthy that minor PIP species with varied fatty acid profiles across different tissues may play crucial roles in physiological processes. The majority of PIPs are of the 1-stearoyl-2 arachidonyl form (*i.e.*, C38:4) with arachidonic acid (C20:4) at the *sn*-2 position of the glycerol backbone (Fig. 1*A*) (3, 4, 5). (C38:4)-containing PIPs are a main source of arachidonic acid for the synthesis of eicosanoid lipid mediator (*i.e.*, prostaglandins and leukotrienes) synthesis *via* phospholipase A2 and cyclooxygenases. How and where precisely the enrichment for C38:4 lipids takes place and what role deacylation-reacylation cycles play in determining the fatty acyl composition of PIPs remains incompletely understood. A large body of work indicates that the acyl chain composition of most phospholipid classes including PI is determined at least in part by the remodeling of lipids made *de novo* by the “Lands cycle” (Fig. 1*A*). This cycle involves a combination of phospholipases A1 and A2 that remove the acyl chains in the sn-1 and sn-2 positions, respectively, and acyl coenzyme A transferases (acyl-CoA) transferases with varying specificity for the acyl-CoA species and lysophospholipid acceptor (6, 7). An alternative hypothesis based on *in vitro* studies proposes the selective conversion of (C38:4)-containing DAG, *via* phosphatidic acid, to CDP-DAG destined for PI synthesis (8). Recent data show that PI synthesized from glucose is initially enriched in shorter and more saturated fatty acyl chains but is then rapidly remodeled towards the preferred C38:4 species. This specific fatty acyl composition of PIPs is then maintained during receptor-stimulated activation of phospholipase C (PLC), that is, an enzyme that cleaves plasma membrane–localized phosphatidylinositol (4, 5)-bisphosphate [PI(4,5)P_2_] (see below) into the signaling molecules DAG and inositol-trisphosphate. This recycling pathway is rapidly stimulated during receptor activation of PLC in cells (1).

**Figure 1:**
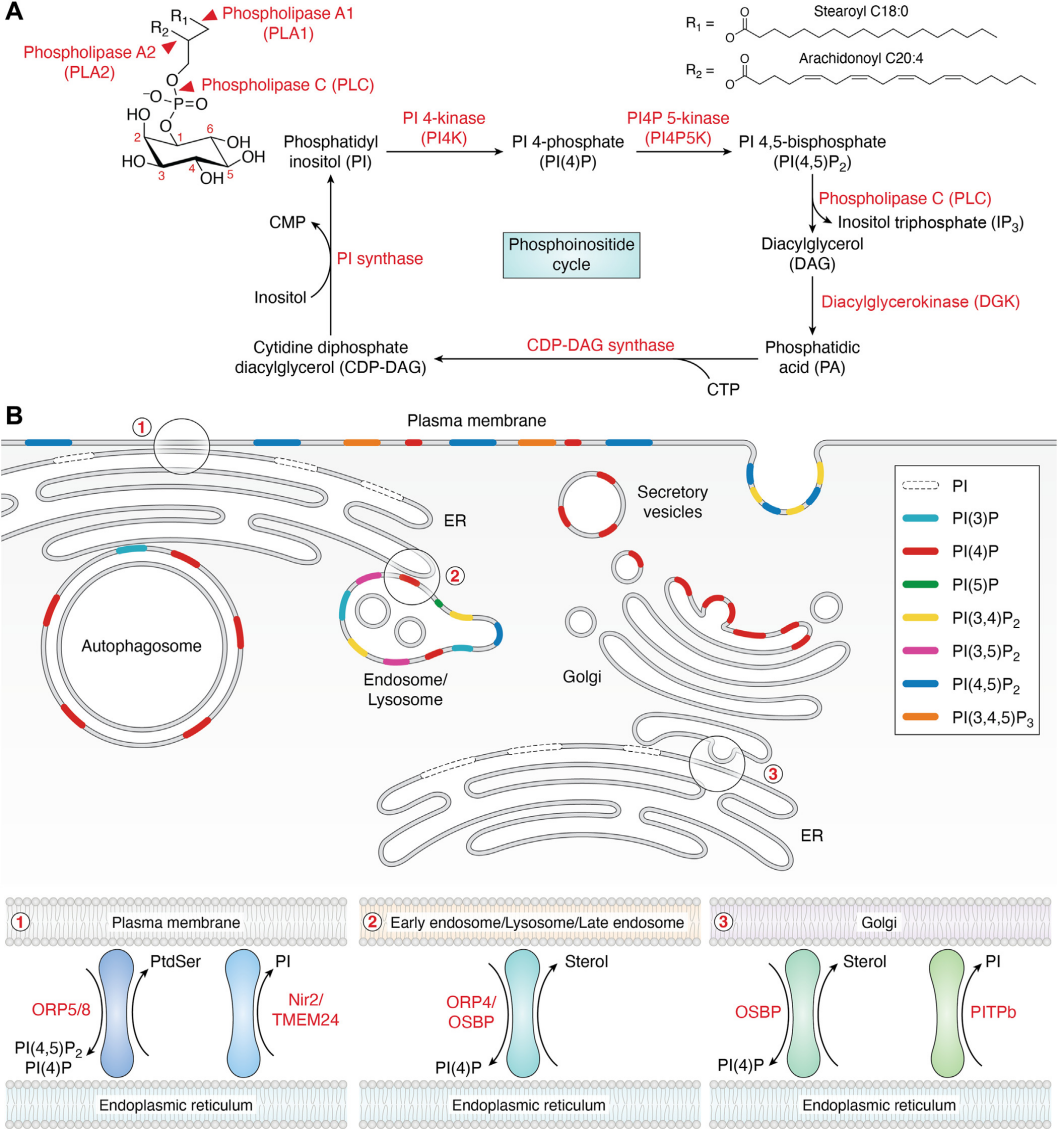
**Phosphoinositide structure, turnover, and localization.***A*, phosphoinositides are glycerophospholipids with a myo-inositol headgroup and two acyl chains (R1 and R2) with the most frequent configuration comprising stearoyl and arachidonyl acyl chains. The acyl chains can be hydrolyzed by phospholipase A1 (PLA1) and phospholipase A2 (PLA2) and the headgroup by phospholipase C (PLC). In the so called phosphoinositide cycle, phosphatidylinositol (PI) is sequentially phosphorylated by PI4-kinase (PI4K) and PI 4-posphate 5-kinase (PI4P5K) to generate PI(4)P and PI(4,5)P_2_, respectively; upon stimulation with receptor agonists, PLC activity hydrolizes PI(4,5)P_2_ into diacylglycerol (DAG) and inositol trisphosphate (IP3); PI generation involves DAG phosphorylation by diacylglycerokinase (DGK) to phosphatidic acid (PA), formation of cytidine diphosphate diacylglycerol (CPD-DAG) by CPD-DAG synthase, and finally exchange of cytidine monophosphate (CMP) for inositol. *B*, PI and its seven phosphorylated derivatives partition into the cytosol-facing leaflets of endomembranes and display non uniform distribution. Lipid transfer proteins use PI(4)P and PI(4,5)P to counter-transport structural lipids (insets 1–3) and supply acceptor membranes with PI that is generated on the ER membrane during the PI cycle (insets 1, 3). ER, endoplasmic reticulum; Nir2, Pyk2 N-terminal domain-interacting receptor 2; OSBP, oxysterol binding protein; ORP, OSBP-related protein; PI(4)P, phosphatidylinositol 4-phosphate; PITPb, phosphoinositide transfer protein beta; PtdSer, phosphatidylserine; PI, phosphatidylinositol; TMEM24, transmembrane protein 24.

While PI constitutes 10 to 20% (mol%) of total cellular phospholipids, its phosphorylated derivatives are much rarer with phosphatidylinositol 4-phosphate [PI(4)P] and PI(4,5)P_2_ as the most abundant PIPs making up for about 0.2 to 1% of total phospholipids. This concentration converts to a density of PI(4,5)P_2_ of 5000 to 20,000 molecules/μm^2^ at the cytoplasmic leaflet of the plasma membrane (9). Other PIPs, for example, such as phosphatidylinositol 3-phosphate [PI(3)P], phosphatidylinositol (3, 4)-bisphosphate [PI(3,4)P_2_], phosphatidylinositol (3, 5)-bisphosphate [PI(3,5)P_2_], and phosphatidylinositol (3,4,5)-bisphosphate [PI(3,4,5)P_3_] are even rarer and may be present at concentrations of less than 10% of that of PI(4,5)P_2_ (1). That said, local enrichment *via* nanoscale localization of PI kinases or phosphatases and their association with specific proteins and/or lipids to spatiotemporally control their distribution may result in much higher concentrations of even rare PIP species at defined intracellular sites. A good example is the local synthesis of PI(3,4)P_2_ *via* class II PI 3-kinase C2α (PI3KC2α) at a rate of four lipid molecules per enzyme per second resulting in a local concentration of PI(3,4)P_2_ within endocytic clathrin-coated pits at the plasma membrane similar to that of PI(4,5)P_2_ (10, 11). The spatiotemporal control of PIP localization and turnover directly impacts on the localization and activity of cytosolic effector proteins and membrane-integral proteins such as receptors, ion channels, or transporters that interact with PIPs. PIP-binding domains of effector proteins such as the well known pleckstrin homology (PH) domains or the PI(3)P-binding Fab1, YOTB, Vac1, EEA1 (FYVE) domain (1, 2) typically associate with their target PIP lipid with a relatively low affinity (*e.g.*, low μM K_d_). As a result, multivalent coincident recognition of additional factors, including small GTP-binding proteins, other lipids, as well as avidity effects caused by the presence of multiple lipid-binding sites or domains on effector proteins, are required to elicit a physiological response, for example, a signaling cascade or assembly of a protein complex that can alter membrane dynamics. Dual key recognition of PIP lipids and proteins (*e.g.*, GTPases) also underlies the observed compartment specificity of PIP-binding domain-based lipid biosensors. This organization enables cells to rapidly adapt and rewire membrane dynamics and cell signaling to altering conditions, for example, signaling responses elicited by hormones or neurotransmitters or alterations in nutrient supply.

The physiological function of PIPs is closely linked to their differential distribution between the various organelles or organellar subcompartments of eukaryotic cells. From a global, somewhat superficial perspective, PI 4-phosphates are concentrated in the secretory pathway with a particular enrichment of PI(4)P in the Golgi complex and of PI(4,5)P_2_ as well as PI(4)P on the cytoplasmic leaflet of the plasma membrane. In contrast, PI 3-phosphates such as PI(3)P and PI(3,5)P_2_ are predominantly found on endosomes, lysosomes, and autophagosomes (2). Finally, PI(3,4,5)P_3_ and PI(3,4)P_2_ represent transient PIP species induced by receptor signaling and/or synthesized during endocytic traffic from the cell surface to endosomes (12).

Progress in studying PIPs is necessarily tied to the development of new biosensors and the diversification of the existing biosensor pool. Genetically encoded PIP biosensors often require proteinaceous-binding partners or specific lipid environments to efficiently report on PIP localization and dynamics. Proper usage and interpretation of the resulting data therefore requires caution. For the interested reader, we refer the reader to excellent recent reviews regarding available PIP biosensors and their limitations (2, 13). Using novel PIP biosensors and imaging techniques, experiments in recent years have challenged the above mentioned simplistic view by suggesting that most PIP species can be found on organellar subdomains under specific circumstances or physiological conditions. For example, lysosomes have been found to contain not only PI(3)P and PI(3,5)P_2_ but have also been associated with PI(4)P, PI(3,4)P_2_, and PI(4,5)P_2_, for example, under conditions of starvation-induced autophagic lysosome reformation (2). These latter findings suggest that PIPs, rather than functioning as signposts of membrane identity, may act as molecular switches that direct the function and fate of a given membrane subcompartment or nanodomain. This switch-like behavior of PIPs enables them to (i) direct the recruitment or assembly of cytoplasmic proteins, (ii) elicit signaling cascades to control cell growth and metabolism, cytoskeletal dynamics, and cell migration, or to (iii) control the activity and assembly status of membrane proteins (*i.e.*, receptors, ion channels) or, in some cases, protein secretion (*e.g.*, of fibroblast growth factor 2, FGF2). The goal of this review is to highlight the molecular mechanisms that turn PIPs into molecular switches, along with their roles in biology as well as their relationship to human diseases.

### PIP switches

As illustrated in Figure 2, four different mechanisms can be defined by which PIPs can be turned on as activated molecular switches, coordinating a range of cellular processes with physiological functions of outstanding importance. As shown in Figure 2*A*, one mechanism by which PIPs can induce a signaling event is based on protein recruitment concomitant with a PIP-induced conformational change that triggers downstream signaling in a membrane-dependent manner. A second way by which PIPs can induce downstream events is their dynamic lateral reorganization into nanodomains that can build platforms for protein recruitment or clustering of divalent cations (Fig. 2*B*). Possible outcomes are PIP-dependent protein oligomerization on membrane surfaces with the potential to trigger membrane remodeling. PIP-containing nanodomain formation is frequently modulated by other membrane lipids, such as cholesterol and sphingomyelin, which are suggested to influence the membrane's compactness and mechanics (14). A third way by which PIPs trigger signaling events is their participation in the dynamic formation of membrane contact sites to enable the exchange of lipids, ions, and small metabolites between organelles (Fig. 2*C*). Finally, through the dynamic action of PIP kinases and PIP phosphatases, a local remodeling of the various PIP species on a membrane surface of a given organelle can produce PIP signatures that provide organelle identity or dynamics (2) coupled to function (Fig. 2*D*). In the following section, we provide examples for each of these mechanisms in depth to illustrate the different mechanisms by which PIPs can act as molecular switches, triggering downstream events that mediate important physiological functions.

**Figure 2:**
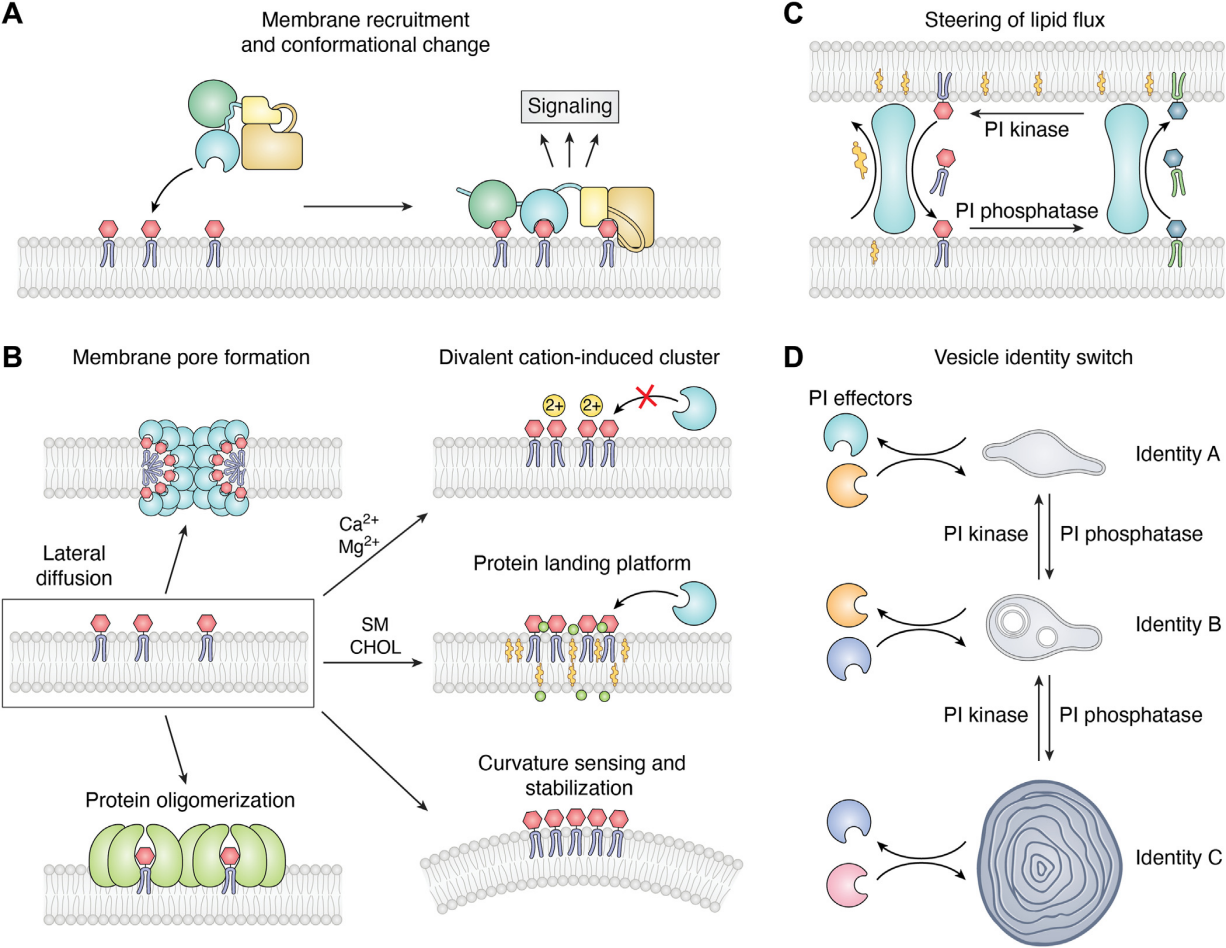
**Modes of PIP switches.***A*, PIP generation on endomembranes may induce recruitment of proteins with specific PIP-binding sites leading to conformational changes and allosteric activation switches. *B*, lateral segregation of PIPs may lead to a membrane pore formation (*upper left*), formation of divalent cation-dependent clusters that prevent protein recruitment (*upper right*), formation of protein landing platforms (*middle right*), membrane curvature sensing and stabilization (*lower right*), or protein oligomerization (*lower left*). *C*, PIPs may induce formation of membrane contact sites and lipid transfer across endomembranes by recruitment of lipid transfer proteins. *D*, sequential phosphorylation and dephosphorylation of PIP species and recruitment of PIP effector proteins may lead to vesicles identity switches. PIP, phosphoinositide.

## PIP-dependent protein recruitment and conformational regulation

Protein kinase B (Akt), with its PH domain, serves as a prime illustration of how phosphatidylinositol phosphate (PIP) synthesis facilitates the attraction and structural activation of proteins. Akt, a central catalyst of cellular growth and a significant contributor to the development of many human cancers, binds explicitly to PI(3,4,5)P_3_ and its downstream hydrolysis product PI(3,4)P_2_. Akt encompasses a group of three serine/threonine-specific protein kinases that play crucial roles in a wide range of cellular functions, including glucose metabolism, programmed cell death (apoptosis), cell proliferation, and migration (15).

**Figure 3:**
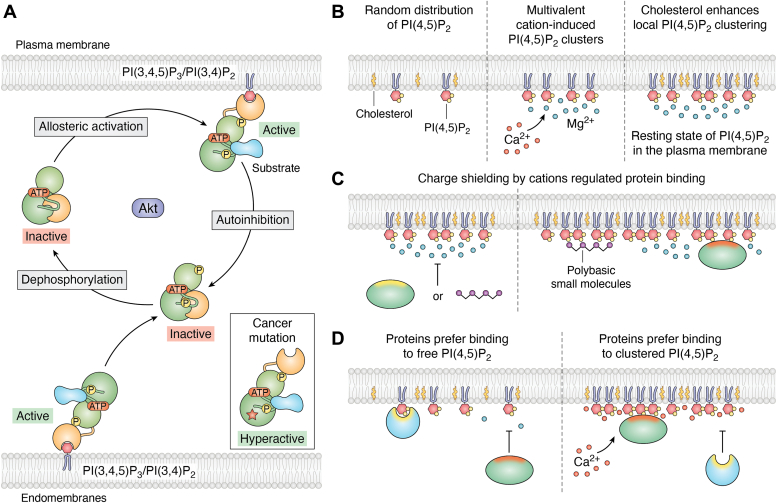
**Interactions between proteins and PIPs.***A*, allosteric activation of Akt by PI(3,4)P_2_ and PI(3,4,5)P_3_. Cytosolic Akt in resting cells is in an autoinhibited conformation and dephosphorylated. PI(3,4)P_2_ and PI(3,4,5)P_3_ recruit Akt to the plasma membrane and endomembranes *via* direct binding to its PH domain (*orange*), which leads to phosphorylation of critical residues, allosteric activation, and substrate binding. Upon dissociation from the membrane, Akt adopts the autoinhibitory conformation and is rapidly dephosphorylated. Mutations prevalent in human cancers prevent adoption of the autoinhibitory conformation and render Akt hyperactive. Adapted from (98). *B*, multivalent cations under physiological conditions promote the formation of PI(4,5)P_2_ clusters, creating a heterogeneous distribution of free and clustered PI(4,5)P_2_ in the plasma membrane. *C*, the interaction of proteins with PI(4,5)P_2_ lipids is influenced by charge shielding and sequestration of negatively charged PI(4,5)P_2_ headgroups by cations and polybasic proteins. *D*, local fluctuations in cation levels may diminish the binding of proteins targeting free PI(4,5)P_2_, while bolstering the binding of proteins targeting clustered PI(4,5)P_2_. *B*–*D*, panels are modified with permission from the Annual Review of Biochemistry, Volume 90 © 2021 by Annual Reviews, http://www.annualreviews.org (32). PH, pleckstrin homology; PI, phosphatidylinositol; PI(3,4)P2, phosphatidylinositol (3, 4)-bisphosphate; PI(3,4,5)P3, phosphatidylinositol (3–,4,5)-bisphosphate; PIP, phosphoinositide.

Akt is recruited to the plasma membrane *via* a specific interaction of its PH-domain with PI(3,4,5)P_3_. Figure 3*A* illustrates that binding to PI(3,4,5)P_3_ triggers a conformational change in Akt, allowing substrate binding. This conformational shift facilitates subsequent phosphorylation by upstream PIP-dependent protein kinase 1 (PDK1) and the mammalian target of rapamycin complex 2. Once activated, Akt proceeds to phosphorylate a spectrum of downstream targets involved in diverse cellular processes such as cell survival, growth, metabolism, and protein synthesis (16). As long as Akt is anchored to PI(3,4,5)P_3_ and remains phosphorylated, it effectively transfers phosphate groups to various substrates. However, when it detaches from PI(3,4,5)P_3_-enriched membranes, Akt switches back to an autoinhibited form, significantly reducing its substrate-binding affinity. Akt is rapidly dephosphorylated in this inactive state, ceasing its catalytic activity (17). Furthermore, the overproduction of PI(3,4,5)P_3_ is associated with the hyperactivation of Akt, leading to a surge in its kinase activity. Such overactivity of Akt can trigger a cascade of critical effects, including metabolic dysregulation, programmed cell death inhibition, and increased cell proliferation and survival. In conclusion, Akt's activity is spatio-temporally regulated by a PI(3,4,5)P_3_-responsive switch. This mechanism ensures that the kinase's activation is confined to cellular regions where PI(3,4,5)P_3_ is located, thus maintaining precise control over Akt's cellular functions. Intriguingly, in the unphosphorylated form, many of the Akt substrates are localized to other subcellular compartments than the plasma membrane, the primary site of PI(3,4,5)P_3_ and PI(3,4)P_2_ generation and, thus, of Akt activity. Prominent examples of such substrates are forkhead box O (FOXO) transcription factors, which localize to the nucleus (18), the mitochondrial Bcl-2 family member Bad (19), and tuberous sclerosis complex 2, a protein localizing to the cytosolic leaflet of the lysosomal membrane (20). Addressing whether the mechanism of Akt substrate phosphorylation comprises dynamic shuttling of Akt substrates between the cytosol and the resident compartment, local activation of Akt at those compartments by minor pools of PI(3,4,5)P_3_ and/or PI(3,4)P_2_, or both, will yield revealing insight into the biology of signaling specificity.

A similar allosteric activation mechanism driven by PIPs has been observed in other kinases such as SGK3 (21) and protein kinase Cs (22), suggesting a common regulatory pattern among different kinases. This underlines the broader relevance of PIPs in the allosteric control of kinase activities beyond Akt, indicating a conserved strategy in cell signaling dynamics.

## Lateral organization of PIPs as a functional switch

PIPs can also be turned into activated molecular switches mediated *via* their lateral reorganization into nanodomains in which they accumulate, as illustrated in Figure 2*B*. The spatial arrangement of PIPs within both living cells and model systems has been thoroughly investigated utilizing a variety of techniques such as fluorescence correlation spectroscopy, fluorescence recovery after photobleaching, atomic force microscopy, and super-resolution microscopy techniques. For example, PI(4,5)P_2_ concentration and distribution in PC12 cells have been investigated using stimulated emission depletion microscopy. This study revealed that PI(4,5)P_2_ exhibits a high concentration within nanoscale membrane domains (23). The tendency of PIPs to aggregate into clusters is influenced by multiple factors, including their interactions with proteins, associations with other lipids (*e.g.*, cholesterol and sphingomyelin), and divalent cations (*e.g.*, calcium and magnesium). These clusters modulate protein–protein interactions influence protein localization and membrane remodeling. The strategic assembly of PIPs imparts a level of structural order and specificity vital for precisely regulating signal transduction, enzymatic activity, and membrane trafficking mechanisms.

### Membrane nanodomain formation and protein recruitment

PIPs have a unique capacity to self-organize in a protein-free environment due to strong electrostatic interactions between their negatively charged headgroups and divalent cations. For example, a single divalent cation can coordinate two PI(4,5)P_2_ lipids at the molecular level, forming strong electrostatic interactions with each lipid's phosphomonoester group. On the other hand, a single PI(4,5)P_2_ lipid can bind up to three divalent cations, thereby interacting with three other PI(4,5)P_2_ lipids (24). This PI(4,5)P_2_–cation interaction pattern can form a densely crosslinked lipid network. Using techniques such as atomic force microscopy and fluorescent analogs of PIPs, clusters have been detected in lipid monolayers with physiological concentrations of calcium and PI(4,5)P_2_ (25, 26, 27, 28). Furthermore, the biophysical properties of this phospholipid within clusters induced by divalent cations are markedly distinct from those of the nonclustered membrane lipids. The engagement of divalent cations causes transformations in the exposure of the headgroup, which in turn results in a reduced solvent-accessible area (29). This process inherently directs the orientation of the headgroup, effectively screening the negatively charged headgroup from binding partners (30). Proteins such as PH-PLCδ1, which possess a typical PI(4,5)P_2_-binding site, are proposed to engage with the PI(4,5)P_2_ headgroup in a one-to-one ratio, suggesting a non-cooperative mode of interaction. In contrast, proteins featuring a polybasic region tend to bind PI(4,5)P_2_ multivalently. For instance, the myristoylated HIV-1 matrix protein shows a preference for binding to divalent ion–induced PI(4,5)P_2_ clusters rather than isolated PI(4,5)P_2_ molecules, a preference not shared by its non-myristoylated mutant variant, which binds more readily to individual PI(4,5)P_2_ molecules (31). These observations lead to the hypothesis that proteins with a high affinity for membranes can overcome the barrier presented by multivalent cations that shield PI(4,5)P_2_ headgroups. Conversely, proteins with lower membrane affinity can only access unshielded, free PI(4,5)P_2_ headgroups, as Figure 3, *B–D* illustrates (32).

In contrast to the dense lipid networks formed by divalent cation-induced PI(4,5)P_2_ clustering, PIP clusters induced by cholesterol exhibit a unique transient behavior. Molecular dynamics simulations have revealed that cholesterol facilitates the organization of PI(4,5)P_2_ molecules into trimeric and tetrameric structures. These structures create areas of elevated negative charge on the membrane surface, which in turn enhances the binding of proteins with specific PI(4,5)P_2_ recognition domains, such as PH-PLCδ1 and FGF2 (33). The specificity of this interaction seems derived from the presence of cholesterol's hydroxyl group. Cholesterol derivatives lacking this group fail to stabilize PI(4,5)P_2_ domains, suggesting that intermolecular hydrogen bonding plays a pivotal role in this process (34).

The interaction between cholesterol and PI(4,5)P_2_ underscores the essential role of cholesterol's hydroxyl group in screening the repulsive interactions among negatively charged PI(4,5)P_2_ molecules. This interaction represents an alternative membrane organization strategy, distinct from the electrostatically driven ionic bridges created by divalent cations.

### PIP-dependent protein oligomerization and membrane pore formation

Following the formation of PIP clusters as outlined above, these lipid domains may serve as potent high-avidity platforms for protein recruitment with high affinity for PIP head groups. Here, PIPs serve as dynamic molecular switches that may facilitate the oligomerization of proteins (35, 36, 37, 38). Such a cascade of interactions could lead to complex membrane structures, including the formation of membrane pores— a crucial step in a broad range of cellular processes.

**Figure 4:**
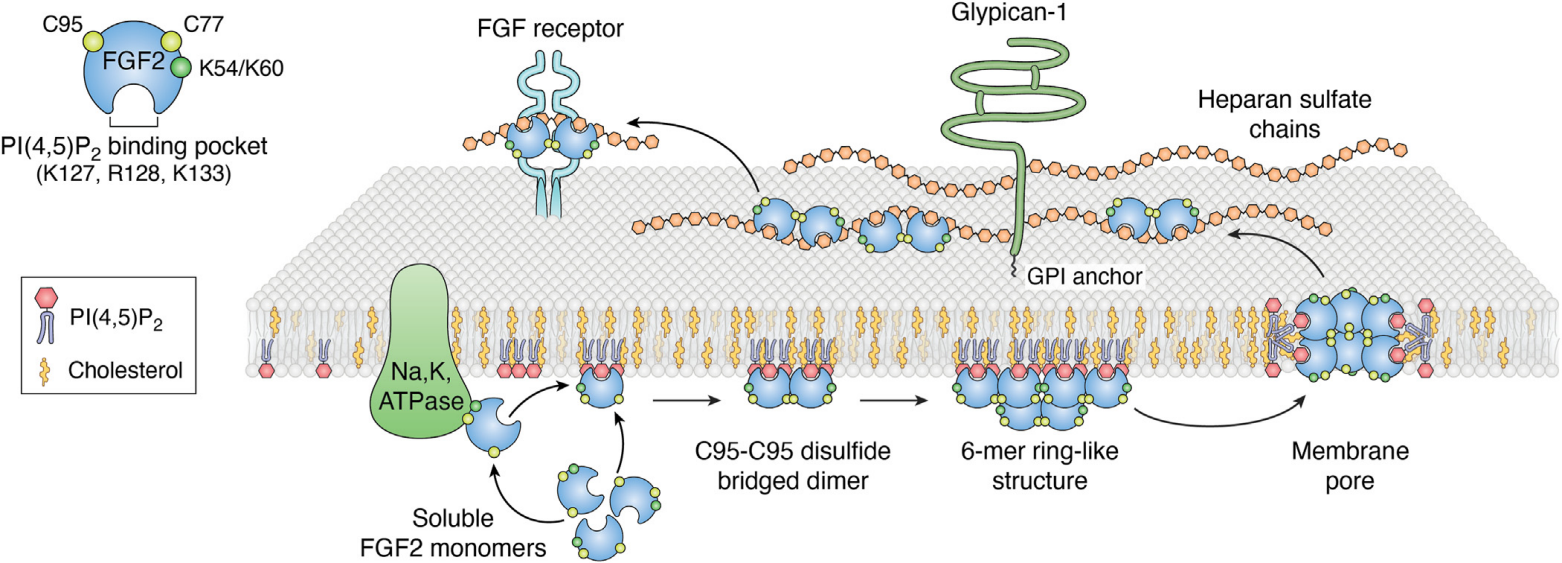
**The unconventional secretory pathway of FGF2.** The secretion of FGF2 is based on a mechanism that occurs *via* direct protein translocation across the plasma membrane. In a first step, soluble FGF2 monomers engage with the inner leaflet of the plasma membrane through a direct interaction with the Na, K-ATPase. Afterward, it is handed over to PI(4,5)P_2_ as part of cholesterol-enriched nanodomains, an interaction that triggers the formation of FGF2 oligomers. As an intermediate of this process, disulfide-bridged FGF2 dimers are formed involving C95. These dimers, along with monomeric FGF2, are thought to self-assemble into hexameric ring structures. The PI(4,5)P_2_-binding pocket of FGF2 is surrounded by multiple basic amino acids that bind an additional four to five PI(4,5)P_2_ molecules through weaker interactions. This process has been proposed to result in a clustering of approximately 30 PI(4,5)P_2_ molecules underneath an FGF2 hexamer. Given that PI(4,5)P_2_ is a non-bilayer lipid, its FGF2-dependent local accumulation was hypothesized to compromise the plasma membrane's integrity, giving rise to a toroidal lipidic membrane pore. The translocation of FGF2 is then finalized when the oligomers are captured and disassembled into dimers, mediated by the cell surface heparan sulfate proteoglycan glypican-1, a process that results in translocation of FGF2 to cell surfaces. FGF2, fibroblast growth factor 2; GPI, glycosylphosphatidylinositol; PI, phosphatidylinositol.

As illustrated in Figure 4, FGF2 is an example of a protein that, upon PIP-dependent membrane recruitment, forms oligomers (35, 39, 40). It is recruited to the inner plasma membrane leaflet *via* the phosphoinositide PI(4,5)P_2_. FGF2 is an unconventionally secreted mitogen exported from cells by direct translocation across the plasma membrane. FGF2 membrane translocation involves the formation of short-lived (200 ms; (41)) lipidic membrane pores. Upon PI(4,5)P_2_-dependent recruitment to the inner plasma membrane leaflet, FGF2 is converted into dimers, followed by the formation of higher oligomers with about 4 to 6 subunits (42, 43). Intriguingly, beyond the high-affinity binding pocket for PI(4,5)P_2_, FGF2 was found to attract 4 to 5 additional PI(4,5)P_2_ molecules through low-affinity interactions (40). Similar findings were reported for other PIP-binding proteins (44). In the case of FGF2, this phenomenon results in an accumulation of about 30 PI(4,5)P_2_ molecules underneath FGF2 hexamers, occupying a membrane surface area of about 10 nm^2^. Since PI(4,5)P_2_ is a nonbilayer lipid, it has been proposed that a local accumulation of PI(4,5)P_2_ molecules may destabilize the plasma membrane lipid bilayer, resulting in the spontaneous formation of lipidic membrane pores with a toroidal architecture (33, 45, 46). This type of membrane remodeling is driven by the thermodynamic stabilization of the high curvature of these pores, mediated by cone-shaped PI(4,5)P_2_ molecules. Upon capturing of FGF2 oligomers by the heparan sulfate proteoglycan glypican-1 at the outer membrane leaflet (47), FGF2 membrane translocation into the extracellular space is completed (47, 48). Thus, PI(4,5)P_2_ can act as a molecular switch triggering FGF2 oligomerization concomitant with membrane remodeling, producing lipidic membrane pores of a highly transient nature that are the basis of this unusual protein secretion pathway.

PIP-dependent oligomerization leading to pore formation appears to be a shared mechanism among specific pore-forming proteins, as exemplified by Gasdermin D, a protein recognized for its role in pore formation (49, 50, 51). Previous studies have shown that negatively charged lipids, mainly PI(4,5)P_2_, play a critical role in Gasdermin D pore formation (51, 52). Furthermore, like FGF2, the oligomerization of Gasdermin D is enhanced by PI(4,5)P_2_ molecules that bridge adjacent monomers, functioning as a molecular 'double-sided tape' that provides additional stability and functionality to these protein assemblies (53). Notably, recent studies have observed an early-stage conversion of PI(4,5)P_2_ to PI(3,4,5)P_3_ during pyroptosis, a form of programmed cell death. This conversion is likely to play a critical role since PI(3,4,5)P_3_ has been linked to stabilizing the Gasdermin D pore structure (54). This insight opens new avenues for mechanisms potentially regulating Gasdermin D–mediated pore opening by manipulating PIP metabolism, offering a strategic approach to modulate pyroptosis in various diseases.

## PIP switches in the control of membrane contact sites

Organellar membranes comprise specialized lipid constituents evolved to support their specialized function. The dissipative nature of organellar membranes and constant flux necessitate the maintenance of the nonuniform lipid distribution. This is achieved by lipid transfer proteins (LTPs) that can shuttle lipids against their concentration gradient and function at membrane contact sites. Both membrane contact sites and the LTPs functioning within these structures are frequently organized by PIPs and their metabolizing enzymes in a switch-like manner (Figs. 1*B* and 2*C*). PIP switches thus allow to establish and to dissolve membrane contact sites in a highly regulated fashion which enables the steering of lipid fluxes according to the signaling or nutrient status of the cell.

Cholesterol levels in the lysosomal limiting membrane, in the secretory pathway, and at the plasma membrane are regulated by OSBP-related protein (ORP) family members. ORPs are LTPs that use PI(4)P to counter-transport cholesterol and other lipids between opposing membranes at membrane contact sites (55). Interestingly, PI(4)P serves dual roles in this process: First as anchor for ORPs at the acceptor membrane and second, as counter-transport metabolite that enables transport of cholesterol against its concentration gradient (56). The turnover of PI(4)P by the ER resident phosphatase Sac1 is thought to provide the energy input for the cholesterol transfer, a mechanism dubbed the “phosphoinositide-motive force” (57, 58). Such an arrangement allows for switch-like steering of cholesterol fluxes by regulating PI(4)P levels.

**Figure 5:**
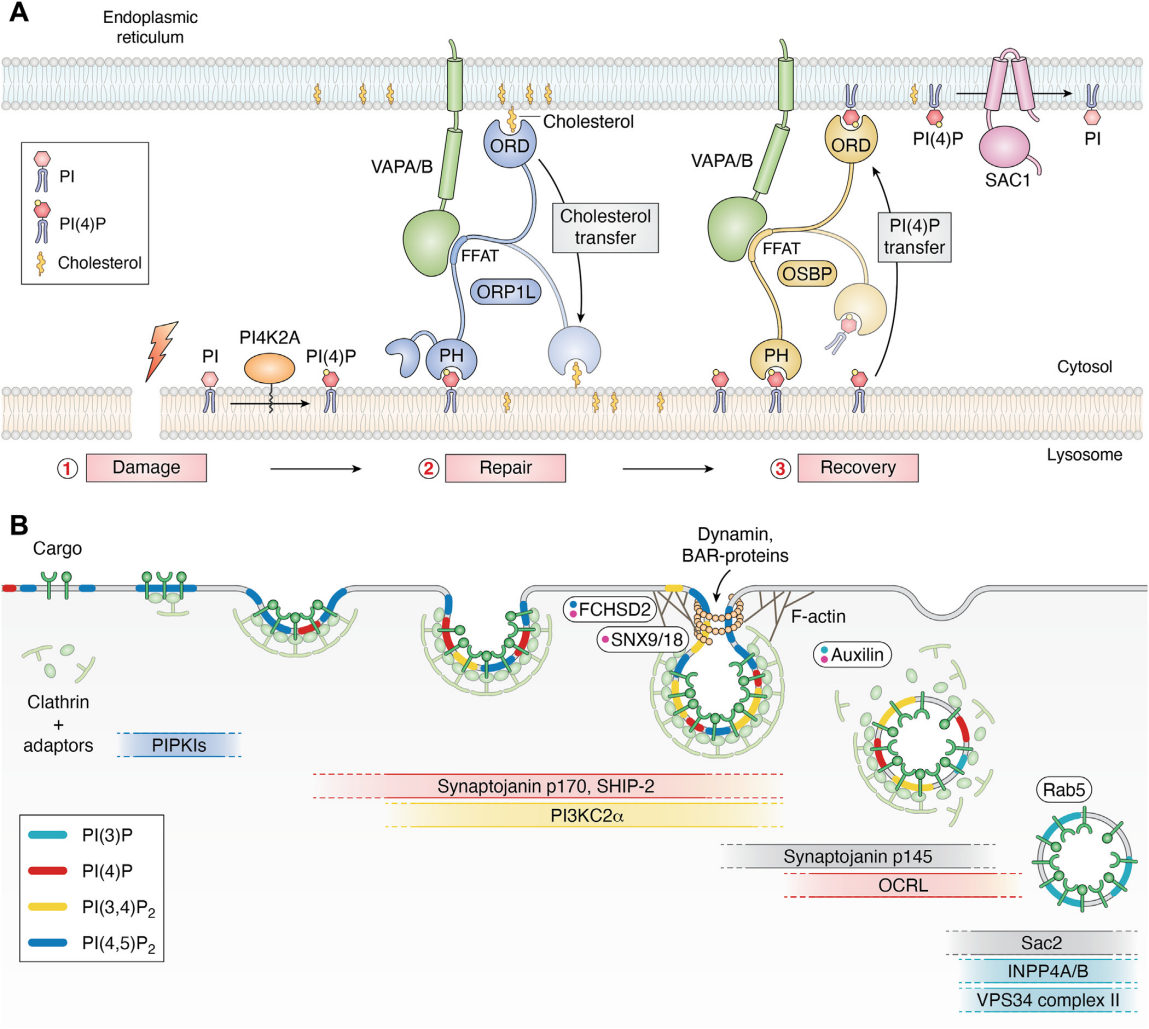
**PIP switches in lysosomal repair and endocytosis.***A*, lysosomal damage induces membrane contact site formation and lipid transfer. Perforation of the lysosomal membrane leads to recruitment of PI4K2A, PI(4)P generation, formation of membrane contact sites between the ER and lysosomes, and lipid transfer *via* ORPs such as ORP1L or OSBP. Adapted from (60). *B*, PIP switches in endocytosis. Endocytic pit assembly requires generation of PI(4,5)P_2_ by phosphatidylinositol (4) phosphate 5 kinase type I (PIPKI). Thereafter, sequential action of PI kinases, PI phosphatases, and PI-binding proteins leads to progressive invagination of the membrane, neck constriction, abscission, and formation of Rab5-positive early endosomes. ER, endoplasmic reticulum; FFAT, two phenylalanines in acidic tract; PH, pleckstrin homology domain; PI, phosphatidylinositol; PI(4)P, phosphatidylinositol 4-phosphate; PIP, phosphoinositide; ORD, OSBP-related domain; ORP, OSBP-related protein; ORP1L, oxysterol-binding protein–related protein 1L.

The switch-like nature of PIP-mediated membrane contact site formation and steering of lipid fluxes is very well exemplified by recently uncovered mechanisms of lysosome repair (59, 60): Perforation of the lysosomal limiting membrane by lysosomotropic agents leads to rapid recruitment of the PI 4-kinase PI4K2A. PI4K2A generates high levels of PI(4)P at the limiting membrane of the damaged lysosomes which induces the formation of extensive membrane contacts between damaged lysosomes and the ER. These membrane contact sites are enriched in LTPs such as ATG2 and ORPs, which transfer structural lipids required for lysosomal repair, for example, phosphatidylserine, cholesterol, and potentially others from the ER to damaged lysosomes (Fig. 5*A*). Additionally, it has been shown that lysosomal cholesterol serves to protect the lysosomal membrane from excessive damage induced by lysosomotropic agents (60). By which mechanism PI4K2A is recruited and/or activated upon lysosomal damage and thus how exactly the switch is induced is unclear. PI4K2A associates with membranes *via* palmitoylated cysteines; however, its localization is under regulation: Earlier biochemical (61, 62) and recent imaging-based work (63) demonstrated that PI4K2A membrane localization is controlled by cholesterol levels. How these findings can be reconciled with the massive recruitment and/or activation of PI4K2A by lysosome damage is unclear. A recent study identified highly conserved serine and threonine residues within the PI4K2A C-terminal tail that are phosphorylated by S6K, a downstream target of mTORC1 (64). Mutation of these residues led to increased localization of PI4K2A to lysosomes and increased PI(4)P production. Intriguingly, lysosome damage causes mTORC1 inhibition, suggesting a possible link between lysosome damage and PI4K2A activation. How exactly lysosome damage engages the PI(4)P switch mechanism will be exciting to see. A so far unexplored possibility is the engagement of phosphoinositide transfer proteins and transfer of PI from the ER to lysosomes to supply PI4K2A with excess substrate. Such a mechanism appears likely as the PI level on lysosomes is very low (65). PI transfer from the ER may also be required to fuel PI(3)P synthesis mediated by the class III phosphoinositide 3-kinase (PI3K) Vps34 on early endosomes to drive the formation of membrane contacts with the tubular ER in fed cells (66). Future research will also have to clarify whether PI(4)P degradation by Sac1 in the ER is a prerequisite for the transfer of cholesterol and other lipids upon lysosomal damage and whether a PIP-motive force or equivalent mechanisms are required for the transport activity of LTPs such as ATG2. Interestingly, ATG2 was found to be a principle component of the phagophore expansion machinery in autophagosome biogenesis and its recruitment was shown to depend on PI(3)P generation by VPS34 (67). These findings suggest that similar PI-dependent switch mechanisms control lysosomal membrane repair and autophagosome biogenesis *via* ATG2-mediated lipid flux. How the transport activity and directionality of LTPs such as ATG2 is regulated and how exactly PIPs engage LTP-dependent lipid transport switches will undoubtedly be fruitful areas of future research.

PIPs themselves are asymmetrically distributed among organelles and require membrane contact sites to maintain their distribution. First, PI(4)P counter-transport from donor membranes to the ER by ORPs necessitates a buffering mechanism that refills PI levels at these membranes. Similarly, PLC signaling activity depletes plasma membrane pools of PI by cleaving PI(4,5)P_2_ into inositol-trisphosphate and DAG. To replenish PI levels, an ancient class of LTPs, the phosphoinositide transfer proteins, engage in membrane contact sites and transfer PI from the ER to acceptor membranes. In case of PLC signaling, the replenishment of plasma membrane PI by Nir2 is rapid and regulated in a switch-like manner by relocalizing Nir2 to ER-PM membrane contact sites (68, 69).

In sum, PIPs are instrumental in establishing membrane contact sites between organelles and in driving directed lipid exchange by LTPs to establish and maintain asymmetric lipid distributions. These PIP-controlled membrane contact sites are instrumental in maintaining the asymmetric distribution of PIPs themselves. Thus, PIPs are at the core of reciprocally organized switching mechanisms that control intracellular lipid distribution in a highly adaptable manner.

## PIP switches in exo-/endocytic membrane dynamics and signaling *via* PI kinases and phosphatases

Spatio-temporally controlled PIP synthesis and turnover *via* tightly regulated PI kinases and phosphatases is a prime mechanism for controlling organellar membrane dynamics, for example, endomembrane maturation, the budding and fusion of vesicles or tubules (Fig. 2*D*), and for cellular signaling downstream of plasma membrane receptors (2).

### PIP-switches in endocytic membrane dynamics

The key PIPs of the plasma membrane are PI(4,5)P_2_ and PI(4)P (70), while PI 3-phosphates dominate the endosomal system (1, 2) (Fig. 1*B*). Hence, for endocytosed cargo to be delivered from the plasma membrane to endosomes, PIP identity needs to be converted from PI(4,5)P_2_ to PI(3)P (Fig. 5*B*). During clathrin-mediated endocytosis, endocytic pits are assembled *via* the association of endocytic proteins such as the early-acting clathrin adaptor AP-2 and the Bin-Amphiphysin-Rvs domain proteins Fer/Cip4 homology domain-only (FCHo) 1/2 (71) with PI(4,5)P_2_. Endocytic pit nucleation and PI(4,5)P_2_ synthesis are feedback-controlled *via* activation of PI(4)P 5-kinase, the major PI(4,5)P_2_ synthesizing enzyme, by AP-2-cargo (internalizing membrane proteins) complexes (72). PI(4,5)P_2_, cargo, and FCHo proteins trigger conformational opening of AP-2 to enable binding to cargo and to clathrin (73, 74). This suggests a feed-forward loop of endocytic adaptors binding to PI(4,5)P_2_ and cargo to trigger increased local formation of PI(4,5)P_2_ during early steps of endocytic vesicle formation. Growth and maturation of endocytic pits are accompanied by the recruitment of PI(4,5)P_2_ 5-phosphatases such as Synaptojanin1 and SHIP2 causing the gradual depletion of PI(4,5)P_2_. In parallel, clathrin recruits the class II PI3K C2α (PI3KC2α) to produce PI(3,4)P_2_ from PI(4)P (75). Depletion of PI3KC2α or enzymatic hydrolysis of PI(3,4)P_2_ stall endocytic pit dynamics and impair the constriction of invaginated endocytic structures. PI(3,4)P_2_ itself promotes membrane constriction by recruitment and conformational activation of the membrane curvature–inducing proteins SNX9/-18 (75) and by stimulating actin polymerization (76). PI(3,4)P_2_ is eventually converted to PI(3)P at the level of endosomes by 4-phosphatases INPP4A and INPP4B (77, 78). During the final stages of endocytic vesicle formation by dynamin-mediated membrane fission, PI(4,5)P_2_ is hydrolyzed by PI(4,5)P_2_ 5-phosphatases such as synaptojanin1 (79) and the occulocerebrorenal syndrome of Lowe (OCRL) protein (80, 81) (see section on disease). Clathrin-mediated endocytosis thus involves the coupled synthesis and turnover of PI(4,5)P_2_ and the concomitant acquisition of 3-phosphoinositide identity that is characteristic of the endosomal system.

Similar cascades of PI kinase and phosphatase activities control other endocytic pathways downstream of signaling receptors such as macropinocytosis, phagocytosis (82), and fast endophilin-mediated endocytosis. The latter is an endocytic route postulated to internalize activated signaling receptors (*e.g.*, the β1-adrenergic or EGF receptors) *via* class I PI3K-mediated synthesis of PI(3,4,5)P_3_ and its subsequent conversion to PI(3,4)P_2_ *via* SHIP1/2, which then triggers membrane recruitment of the PI(3,4)P_2_-associated actin modulator lamellipodin and, thereby, of the membrane-deforming N-BAR domain protein endophilin to the leading edge of cells (83), from where receptors are internalized into tubular endosomes.

Following internalization, endocytic vesicles coalesce in early endosomes from where cargo can either be recycled to the plasma membrane, trafficked retrogradely to the trans-Golgi network, or get sorted into late endosomes and multi-vesicular bodies for lysosomal degradation. The signature PIP of the endosomal compartment is PI(3)P, which is predominantly synthesized from PI by the sole class III PI3K, VPS34, with cell type–dependent contributions from class II PI3Ks (2, 12).

### PIP-switches in exocytosis

A conceptually similar PIP switch controls the final step of the secretory pathway, that is, vesicle exocytosis from internal compartments to the plasma membrane (Fig. 1*B*). Constitutive exocytosis requires the exocyst, an eight-subunit complex that tethers exocytic vesicles to the plasma membrane. Engagement of the complex with exocytic vesicles is regulated by the small GTPase Rab11 and PI(4)P, a hallmark lipid of trans-Golgi network–derived secretory vesicles. The exocytosis of recycling vesicles that emanate from endosomes marked by PI(3)P requires PI(3)P-to-PI(4)P conversion by the PI 3-phosphatase myotubularin 1 (MTM1) (84), an enzyme implicated in X-linked centronuclear myopathy in humans (see section on disease), and the PI 4-kinase PI4K2A. Interaction of exocyst with the acceptor compartment, that is, the plasma membrane, depends on PI(4,5)P_2_ (85).

Elegant experiments have also revealed a stringent requirement for plasma membrane PI(4,5)P_2_ in regulated exocytic fusion of secretory granules and synaptic vesicles (SVs) in neuroendocrine cells and in neurons. Fast capacitance measurements showed that overexpression of PI(4,5)P_2_ generating or degrading enzymes increased or decreased the readily releasable pool of SVs, respectively (86, 87). Most strikingly, uncaging of photoactivatable membrane-permeant variants of PI(4,5)P_2_ potentiated exocytosis by triggering the rapid fusion of a subset of readily-releasable vesicles. Further genetic experiments identified Synaptotagmin-1, a calcium binding and sensing SV-resident transmembrane protein and the active zone priming protein Munc13 as the PI(4,5)P_2_ effector proteins in neuroexocytosis (88). How exactly PI(4,5)P_2_ binding to Synaptotagmin-1 promotes rapid SV fusion is uncertain. Possible mechanisms involve synergistic interactions of Synaptotagmin-1 with calcium (89, 90, 91, 92), trans interactions with PI(4,5)P_2_ on the plasma membrane to trigger membrane buckling (93, 94), and/or association with the soluble NSF attachment protein receptor complex (95, 96). Interestingly, recent data have uncovered an independent function of exocytosed Synaptotagmin-1 on the plasma membrane in the activity-dependent synthesis of PI(4,5)P_2_ to promote compensatory endocytosis at synapses in mammalian neurons (97). This mechanism serves to couple exocytic vesicle fusion to endocytic membrane internalization to maintain presynaptic membrane homeostasis.

### PIP-switches in cell signaling

Class I PI3Ks catalyze the conversion of PI(4,5)P_2_ to PI(3,4,5)P_3_ by phosphorylating the inositol ring. Based on the differential usage of regulatory subunits, class I PI3K catalytic subunits are divided into two subclasses – IA and IB. Class IA PI3Ks are heterodimers of one of three catalytic subunits (the PIK3CA gene product p110a, the PIK3CB product p110b, or the PIK3CD product p110d), tightly bound to one of the five regulatory subunits (the PIK3R1 gene products p85a/p55a/p50a, the PIK3R2 product p85b, or the PIK3R3 product p55g). p110a and p110b are widely expressed, with p110d predominantly found in leukocytes. P110a signals downstream of plasma membrane–associated tyrosine kinases to RAS and to various other effectors including the Akt oncogene and further downstream to lysosomal mTORC1, an important metabolic control point and driver of cell growth and anabolism. As explained above, binding of PI(3,4,5)P_3_ or PI(3,4)P_2_ to the PH domain allosterically activates Akt to ensures that its activity is restricted to the membrane (98). *Via* these mechanisms, class IA PI3K-mediated conversion of PI(4,5)P_2_ to PI(3,4,5)P_3_ triggers a significant shift in the landscape of the cell by altering the recruitment and activation of proteins that have an affinity for PI(3,4,5)P_3_. Conversely, phosphatase and tensin homolog can restore PI(3,4,5)P_3_ to its PI(4,5)P_2_ state through dephosphorylation, thereby reverting the lipid's role (99, 100). As stated above, PIPs can also be degraded *via* PLC-mediated cleavage into IP_3_ and DAG (99) thereby effectively serving as a shutdown switch for their functions.

In addition to the role of class I–derived PI(3,4,5)P_3_ in the activation of Akt and mTORC1 in insulin- or growth factor–stimulated cells, PIPs also play a local role in regulating mTORC1 signaling at the level of the lysosome. While PI(3)P production by the class III PI 3-kinase VPS34 upregulates mTORC1 and induces lysosome dispersion (64, 101), local generation of a lysosomal pool of PI(3,4)P_2_ by class II PI3K C2β (PI3KC2β) plays an opposing role. In growth factor–deprived conditions, PI3KC2β is recruited to mTORC1 on lysosomes and phosphorylates PI(4)P to generate PI(3,4)P_2_. Local PI(3,4)P_2_ synthesis *via* spatiotemporally controlled recruitment of PI3KC2β promotes the net retrograde transport of lysosomes towards the microtubule-organizing center and represses mTORC1 signaling *via* recruitment of inhibitory 14-3-3 proteins (102) and by facilitating the oxysterol-binding protein–related protein 1L (ORP1L)-mediated transport of cholesterol, an important cofactor for mTORC1 activation, to the ER (103).

These examples illustrate how spatiotemporally controlled recruitment of PI kinases and phosphatases steers intracellular membrane dynamics and regulates cell signaling. Many other pathways (*e.g.*, autophagy) rely on similar PI kinase– and phosphatase-dependent mechanisms to control cell physiology.

## Understanding phosphoinositide switches in human disease

Research over the past decades has identified a growing list of human diseases associated with dysregulation of PIP switches. In most cases, the underlying cause is aberrant cellular levels of specific PIPs due to mutations in specific PIP kinase or, more frequently, phosphatase isoforms. These range from neuromuscular, skeletal, and kidney disorders caused by loss-of-function of PIP phosphatases (*e.g.*, myotubularin family members, FIG4, OCRL) to overgrowth syndrome, immune deficiency, and cancer as a result of hyperactivation of the class I PI3K/AKT signaling pathway. It should be noted that, in many cases, clinical phenotypes overlapping with those caused by mutations in PIP metabolizing enzymes can be induced by mutations in diverse sets of other genes. Often, the relationship of these genes to the pathways controlled by PIP metabolizing enzymes remains unclear.

Instead of providing a comprehensive overview of known PIP-related human diseases ((1, 2, 104) for recent reviews on the topic), we focus here on select examples that illustrate the different mechanisms of how defective PIP switching can lead to disease.

### Diseases involving altered protein recruitment and conformational activation by PIPs—the example of the PI3K–AKT pathway

Arguably, the best-understood examples of human diseases related to PIP-induced protein recruitment and conformational changes are activating mutations in class IA PI3K in cancer (12, 105, 106), in PI3KCA-related overgrowth spectrum, and in activated PI3K-delta syndrome (APDS), a primary immunodeficiency disorder (107).

**Figure 6:**
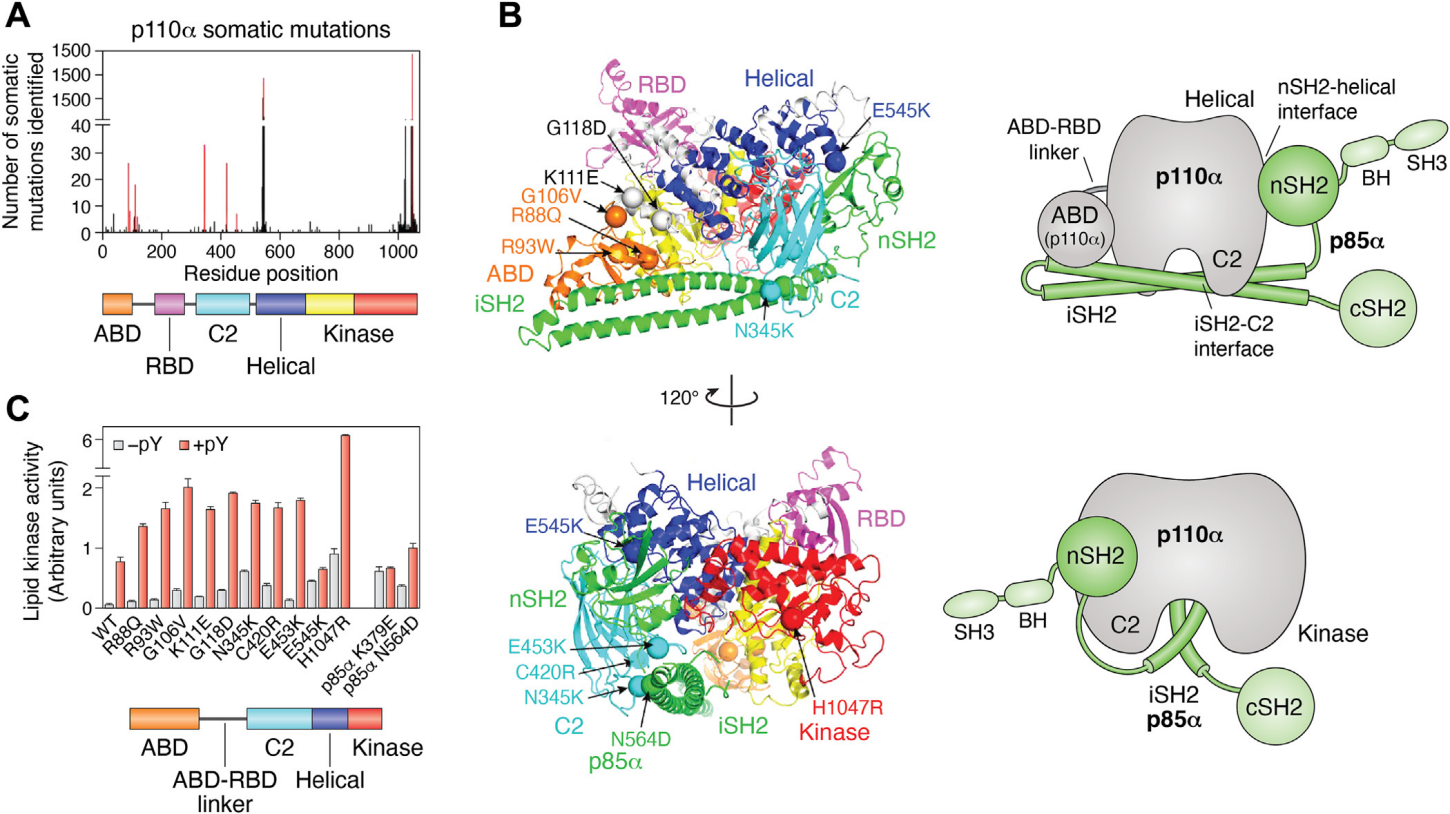
**Disease mutations in p110α.***A*, frequency of somatic mutations in p110α *versus* residue number, with domains of p110α colored. Bars for mutations analyzed in this study are highlighted in *red*. *B*, locations of cancer-linked mutants analyzed in this study are shown as *spheres* mapped on the crystal structure of p110α/niSH2-p85α (Protein DataBank, pdb: 3HHM). Schematics of the crystal structure with catalytic subunit in *gray* and p85 in *green* simplify the views presented. The p85 domains missing from the crystal structure are outlined in *green*. *C*, lipid kinase assays of somatic mutants. Assays measured 32P-PIP3 production. Assays were performed in duplicate and repeated twice. Adapted from (108). PIP, phosphoinositide.

Somatic mutations in *PIK3CA* encoding for the catalytic subunit of the ubiquitously expressed class I PI3Ka isoform p110α occur in up to 30% of some types of common epithelial cancer, including breast, colon, prostate, and endometrial cancers and typically result in hyperactivity of the enzyme and elevated synthesis of its major product PI(3,4,5)P_3_. Under normal physiological conditions, p110a activity is tightly constrained *via* autoinhibition by intramolecular and intermolecular determinants, resulting in transient and localized PI(3,4,5)P_3_ generation. Elegant structural studies (108) have provided insight into the mechanisms by which distinct mutations (Fig. 6*A*) found in different cancers can activate p110α (Fig. 6*B*). Mutations in the linker between the adapter-binding domain (ABD) and the Ras-binding domain (G106V and G118D) of p110α commonly found in endometrial cancers enhance the natural activation *via* movement of the ABD and ABD–Ras-binding domain linker relative to the rest of the catalytic subunit, thereby breaking the C2–iSH2 inhibitory interface. The C2 domain mutants N345K and C420R also mimic these events, even in the absence of membranes. The helical domain mutation E545K causes PI3KCA activation by breaking the nSH2–helical domain contact caused by phosphotyrosine-motif peptides that normally bind and activate the enzyme under physiological conditions. Finally, the interaction of the C lobe of the kinase domain with membranes is potentiated by kinase domain mutations, most notably H1047R (Fig. 6*B*). H1047R is also the most common somatic mutation found in patients suffering from PROS, while other mutations (*e.g.*, E545K) have also been reported. Consequently, these mutations increase lipid binding and basal activity of p110α (Fig. 6*C*), thereby leading to enhanced PI(3,4,5)P_3_ production that contributes to oncogenesis.

APDS is a primary immunodeficiency resulting from increased activity of the class IA PI3Kd isoform that is caused by mutations in the PIK3CD gene product p110d (APDS1) or in the gene encoding the PI3Kd regulatory subunit p85α (APDS2) (109).

To date, more than 10 activating missense mutations have been reported in the PI3KCD gene, resulting in APDS1, with the E1021K variant in the C lobe being the most frequently reported. In the p110d enzyme, E1021K is thought to increase kinase activity *via* a mechanism similar to the well-understood H1047R mutation in p110α described above. Several mutations in the *PIK3R1* encoding p85a have been reported that lead to APDS2. Many of these affect splice sites that lead to the skipping of exon 11 and the in-frame deletion of 42 amino acids within the iSH2-coiled-coil domain of p85α. Consequently, inhibitory interactions with p110d, but surprisingly, not with p110α are disrupted. This effect explains why the phenotype is largely restricted to p110d-expressing immune cells.

Finally, mutations in oncogenic AKT kinases, some of the best-established common effectors of class I PI3K signaling, have been found to occur in 3 to 5% of all human cancers. AKT is an important signaling hub with well over 100 downstream target substrates, affecting cell metabolism, growth, survival, and proliferation. For example, AKT promotes glucose uptake and the storage of energy in the form of glycogen through its effects on glycogen synthase kinase-3. Highlighting these important roles, an inactivating mutation in AKT was found to underlie severe familial insulin resistance and diabetes (110). On the contrary, the activating hotspot mutation E17K within the PH domain, which enhances AKT binding to membranes, promotes cellular transformation and induces leukemia in mice by uncoupling the allosteric activation of AKT1 from binding to PI(3,4,5)P_3_. Interestingly, D323 and D325, two residues also mutated in cancer, are part of the autoinhibitory interface that renders AKT quiescent in the cytoplasm (Fig. 3) (98).

Together, these examples illustrate how aberrant conformational activation of enzymes by PIPs (*i.e.*, PI(3,4,5)P_3_) can lead to tissue overgrowth, immunodeficiency, or cancer and highlight how exquisitely tight control exerted by PIPs on Akt activity is required for homeostatic balance.

### Defective cell signaling and membrane dynamics caused by imbalance of PI kinase and phosphatase activities

The subcellular activities of PIPs rely on the finely tuned nanoscale localization and activities of PI kinases and phosphatases. An imbalance of these activities, most frequently due to loss or reduction of PI phosphatases, results in often severe cell physiological defects (104).

Loss of the 3-phosphatase ‘phosphatase and tensin homolog,’ the enzyme that converts PI(3,4,5)P_3_ to PI(4,5)P_2_, similar to hyperactivation of PI3KCA, has been found in a large variety of human cancers (111), further highlighting the role of PI(3,4,5)P_3_ as an oncogenic lipid. Similarly, loss or inactivation of the PI(4,5)P_2_-metabolizing 5-phosphatase OCRL leads to Lowe syndrome (112) and the milder Dent's disease (*i.e.*, Dent-2) as a consequence of defective endocytic membrane dynamics caused by the accumulation of its main substrate PI(4,5)P_2_. Lowe syndrome is characterized by renal Fanconi syndrome (*i.e.*, a rare kidney disease), dense congenital cataracts, central hypotonia, and mental retardation. More than 130 clinically relevant mutations of the OCRL gene have been identified in Lowe and Dent-2 patients. Missense mutations are commonly found in the 5-phosphatase, ASH (ASPM, SPD2, and Hydin), and RhoGAP domains. They disrupt either OCRL catalytic activity or its correct folding and its ability to interact with some of its partners. In all cases of Lowe syndrome, independently of the mutated site, the ability to dephosphorylate PI(4,5)P_2_ is severely impaired (104, 113).

Further prominent examples of defective PIP switching underlying human diseases are neuromuscular disorders such as Charcot-Marie-Tooth disease (CMT) and centronuclear myopathy. These are most frequently caused by mutations in the myotubularin family of 3-phosphatases that comprises 15 members in total, 9 of which encode active phosphatases that can associate with inactive members in specific combinations thought to define their subcellular location and fine-tune activity. Mutations in MTM1, the founding member of the myotubularin family, cause X-linked centronuclear myopathy (XLCNM), a severe congenital myopathy characterized by muscle weakness and disorganization, among other muscle and non-muscle symptoms. XLCNM patients display a severe variation in muscle fiber size due to the presence of small, round fibers with centrally placed nuclei as well as abnormalities of the muscle triad (114, 115). Among the molecular defects associated with XLCNM are the accumulation of its main substrate PI(3)P, reduced active integrin levels on the cell surface, somewhat variable and possible indirect defects in autophagy, disorganization of mitochondria, and structural defects in the organization of the sarcoplasmic reticulum, the specialized form of the ER in skeletal muscle tissue. Defective myotube formation in XLCNM patients is likely caused by defective recycling of active b-integrins to the cell surface (116, 117), a phenotype that is antagonized by concomitant loss of inhibition of PI 3-kinase C2b in cell and animal models (118, 119, 120, 121). These exciting data suggest that specific pharmacological inhibition of PI 3-kinase C2b may be a viable treatment option for XLCNM patients.

Mutations in the MTMR2 gene cause a different neuromuscular phenotype, the genetic cause of CMT4B1, a demyelinating form of CMT with autosomal recessive inheritance characterized by early onset proximal and distal limb muscle weakness that is often followed by systemic symptoms such as cataracts and deafness. MTMR2 is a ubiquitously expressed catalytically active 3-phosphatase, which *in vitro* dephosphorylates PI(3,5)P_2_ with preference over PI(3)P. A hallmark of CMT4B1 neuropathy are redundant loops of myelin in the nerve termed myelin out-foldings, which can be considered the consequence of altered growth of myelinated fibers during postnatal development. Mechanistic studies have shown that MTMR2 acts as an obligatory heterodimer with the catalytically inactive MTMR5 or MTMR13 pseudophosphatases downstream of the small GTPase Rab35 to promote hydrolysis of PI(3,5)P_2_ in Schwann cells. Mutations in MTMR13 and MTMR5 underlie the closely related CMT types 4B2 and 4B3, respectively. Mutational inactivation of any of the above factors, that is, MTMR2, MTMR5, MTMR13, or Rab35 leads to the accumulation of PI 3-phosphates, most notably PI(3,5)P_2_, and the concomitant hyperactivation of mTORC1 signaling leading to focal hypermyelination. Consistently, it has been found that mTORC1 inhibition and, more potently, pharmacological inhibition of PI(3,5)P_2_ synthesis *via* apilimod ameliorate CMT4B phenotypes (122, 123).

These examples show how rebalancing of PIP levels to restore their switch function may serve as a potential therapeutic avenue to cure human disease.

### Dysregulation of membrane contact sites caused by defective PIP switching

Another disease mechanism related to defective PIP switching includes alterations in the expression levels or function of proteins that regulate lipid transport at membrane contact sites (124). For example, Nir2 (also known as PITPNM1), a protein involved in the nonvesicular exchange of ER-bound phosphatidylinositol (*e.g.* the substrate for PI(4,5)P_2_ synthesis at the plasma membrane) for phosphatidic acid between the ER and the plasma membrane, is overexpressed in certain types of cancers. Overexpression of Nir2 is linked to epithelial-mesenchymal transition in mammary epithelial and breast cancer cells and may contribute to breast cancer progression in human patients (125).

Another example is the PI 3-phosphatase MTM1 (see also above), that is, the enzyme mutated in XLCNM (126, 127), a disease characterized by multiple organellar defects in muscle tissue, including alterations in the morphology of the ER. Recent work shows that MTM1 is part of an organellar conveyor's belt, in which endosomal PI(3)P hydrolysis by MTM1 in response to amino acid starvation (*e.g.*, a frequent condition in metabolically active tissues) causes the reshaping of the peripheral ER from tubules to sheets. As the tubular ER plays a key role in mitochondrial division, this leads to hyperfusion of mitochondria into large networks more than 10-times the size of those observed in MTM1 loss-of-function models (66).

A final example of how PIP switches may underlie human disease is exemplified by PI4K2A. Recent work shows that biallelic PI4K2A variants resulting in PI4K2A deficiency underlie developmental encephalopathy with epilepsy and hyperkinetic movement disorders (128). At the molecular level, the Arg309Ter variant failed to produce PI(4)P at late endosomal/lysosomal membranes. As explained above, PI4K2A plays essential roles in lysosomal repair *via* lipid transport at membrane contact sites with the ER (59, 60) and in promoting lysosomal catabolism in response to starvation (64). It thus seems tempting to speculate that lysosomal dysfunction underlies developmental encephalopathy in patients carrying PI4K2A loss-of-function mutations. The conversion of nutrient-signaling active anabolic to degradative catabolic lysosomes in starved cells in addition to PI4K2A also involves hydrolysis of lysosomal PI(3)P by the PI 3-phosphatase MTMR14 (64), another enzyme mutated in an autosomal form of myotubular myopathy (129), suggesting that altered lysosome function may contribute to inherited forms of myotubular myopathy.

These examples illustrate yet another mechanism based on altered membrane contact sites dynamics by which perturbation of PIP switches may contribute to human disease.

## Future perspectives

In this review, we highlighted various kinds of molecular mechanisms by which PIPs can serve as molecular switches, coordinating a broad range of downstream processes with key functions in cellular physiology. A common denominator of the examples provided appears to be dysregulation of the cellular levels of specific PIP species in a manner highly constrained spatially at defined subcellular locations. The underlying cause is typically the emergence of mutant forms of the various isoforms of PIP kinases and, more frequently, phosphatases, respectively. Therefore, a critical aspect of future drug development will be exploring whether information on the identity of the specific PIP switch can guide the development of drugs for highly targeted therapies that address the dysregulated mechanism with precision.

## Conflict of interest

The authors declare that they have no conflicts of interest with the contents of this article.
